# The Shedding of CD62L (L-Selectin) Regulates the Acquisition of Lytic Activity in Human Tumor Reactive T Lymphocytes

**DOI:** 10.1371/journal.pone.0022560

**Published:** 2011-07-28

**Authors:** Shicheng Yang, Fang Liu, Qiong J. Wang, Steven A. Rosenberg, Richard A. Morgan

**Affiliations:** Surgery Branch, Center for Cancer Research, National Cancer Institute, National Institutes of Health, Bethesda, Maryland, United States of America; Saint Louis University School of Medicine, United States of America

## Abstract

CD62L/L-selectin is a marker found on naïve T cells and further distinguishes central memory (Tcm, CD62L+) from effector memory (Tem, CD62L−) T cells. The regulation of CD62L plays a pivotal role in controlling the traffic of T lymphocytes to and from peripheral lymph nodes. CD62L is shed from the cell membrane following T cell activation, however, the physiological significance of this event remains to be elucidated. In this study, we utilized *in vitro* generated anti-tumor antigen T cells and melanoma lines as a model to evaluate the dynamics of CD62L shedding and expression of CD107a as a marker of lytic activity. Upon encounter, with matched tumor lines, antigen reactive T cells rapidly lose CD62L expression and this was associated with the acquisition of CD107a. By CD62L ELISA, we confirmed that this transition was mediated by the shedding of CD62L when T cells encountered specific tumor antigen. The introduction of a shedding resistant mutant of CD62L into the tumor antigen-reactive T cell line JKF6 impaired CD107a acquisition following antigen recognition and this was correlated with decreased lytic activity as measured by ^51^Cr release assays. The linkage of the shedding of CD62L from the surface of anti-tumor T cells and acquisition of lytic activity, suggests a new function for CD62L in T cell effector functions and anti-tumor activity.

## Introduction

The trafficking of leukocytes from the peripheral blood to the surrounding tissue is pivotal for inflammatory responses and is tightly regulated by cell adhesion molecules on T lymphocytes and endothelial cells [1]. The selectins are a family of cell adhesion molecules including E-selectin, P-selectin and L-selectin, which coordinately contribute to leukocyte tethering and rolling along the luminal surface of venules [2], [3]. L-selectin, also known as CD62L, is a cell adhesion molecule found on leukocytes including lymphocytes, neutrophils, monocytes, eosinophils, and hematopoietic progenitor cells. When a CD62L+ T cell enters a lymph node it can become activated by antigen presenting cells (APC) and upon activation, CD62L is cleaved at K283-S284 by a disintegrin and metalloprotease ADAM17 [4], [5], [6]. Following the shedding of CD62L from antigen-activated T cells in lymph nodes, T cells can reenter the circulation where they can exert their helper or effector functions [7].

A CD62L knockout mouse model indicates that CD62L plays an essential role in lymphocyte homing to lymphoid tissues and sites of inflammation [8]. However, transgenic mice over-expressing a shedding resistant CD62L demonstrated that shedding of CD62L is not required for memory T cell recirculation and homing to peripheral lymph nodes or migration to sites of infection, however, the ability to clear virus was significantly compromised [9]. These authors argued that the delayed viral clearance in this mouse model likely resulted from improper adhesion and migration of lymphocytes [9].

In T cell mediated immune responses, following encounter with pathogens T cells experience an expansion and then a contraction phase, which leads to a long-lived pool of memory cells. Memory T cells can be further classified by their differentiation markers (CD62L and CCR7) or the release of cytokines (IL2 and IFN-γ) as central memory cells (Tcm, CD62L+) and effector memory (Tem, CD62L−) [10], [11], [12], [13]. While there exists heterogeneity for CCR7 [14] marker expression in memory T cells [15], [16], [17], CD62L is more uniformly expressed and a reliable surface protein marker to distinguish between Tcm and Tem. Memory T cell subsets have distinct migratory patterns after encountering antigen and activation. Tem cells primarily migrate to non-lymphoid tissues and inflammation sites, whereas Tcm cells have the capacity to migrate to peripheral lymph nodes [18], [19]. Tcm cells display a capacity for self-renewal, and have been reported in animal studies to lead to sustained engraftment and potent anti-tumor efficacy in adoptive immunotherapy [20], [21]. Tem have a lower proliferative capacity [22], [23], but have a pronounced capacity for immediate effector function mediated by the pre-expression of granzyme B and perforin within intracellular cytotoxic granules, a type of secretory lysosome [10], [24], [25], [26]. These cytoxic granules are often studied by the expression of surrogate marker proteins know as lysosomal-associated membrane proteins. Lysosomal-associated membrane proteins CD107a (LAMP1) and CD107b (LAMP2) are highly glycosylated proteins and predominantly expressed intracellularly in the lysosomal/endosomal membrane in nearly all cells, yet their exact function remains unknown [27]. In T cells and NK cells they are found on the intracellular cytotoxic granules, but not on the cell surface. When the cytotoxic granules transiently traffic to the cell surface during the formation of the immunological synapse and degranulation, this results in the expression of CD107 on the cell surface [26], [28], [29]. CD107a in particular has been described as a marker of NK and CD8+ T-cell degranulation following stimulation [30], [31], [32].

In this study, we used human PBL or purified CD8+ T cells genetically engineered with an anti-tumor TCR [33], [34], [35] as a model to evaluate the dynamics of CD62L regulation after encountering tumor antigen. Using this model, we illustrated, for the first time, that the shedding of CD62L was tumor antigen specific, and coupled to the appearance of a degranulation marker CD107a [31]. Interestingly, the relationship of CD62L shedding and formation of CD107a was reciprocal, i.e., introduction of shedding resistant mutant of CD62L onto surface of CD8+ T cells adversely affected CD107a cell surface expression after encountering tumor antigen.

## Materials and Methods

### Cell culture

All PBL and melanoma cell lines used in this study were obtained under written informed consent from healthy donors and metastatic melanoma patients seeking treatments at Surgery Branch, National Cancer Institute (NCI). All protocols were reviewed and approved by the Institutional Review Board of the National Cancer Institute, Bethesda, MD. Briefly, PBL were collected by leukapheresis, and lymphocytes were separated by Ficoll/Hypaque cushion centrifugation, washed in HBSS and resuspended at a concentration of 1×10^6^/ml in AIM-V medium (Invitrogen, Carlsbad, CA) supplemented with 300 IU/ml IL-2 (Chiron, Emmerville, CA) and 5% heat-inactivated human AB serum (Valley Biomedical, Winchester, VA). Melanoma cell lines were produced at the Surgery Branch NCI from patient material obtained as described above and included MART-1 positive HLA-A*0201^+^ 624, 526 and one MART-1 negative HLA-A*0201^−^ line 938 (all lines available upon request). 293T (ATCC, Manassas, VA) cells were cultured in DMEM supplemented with 10% FCS, 100 U/ml penicillin/streptomycin, 2 mM L-glutamine, 20 µM 2-mercaptoenthanol and 25 mM HEPES buffer solution (Invitrogen). JKF6 is a long-term cultured tumor infiltrated lymphocytes (TIL) from melanoma patient maintained at Surgery Branch NCI. All cell lines were cultured at 37°C in a 5% CO_2_ humidified incubator.

### Vector construction, preparation, and transduction

All lentiviral constructs utilized were derived from pRRLSIN.cPPT.MSCV/GFP.wPRE harboring a green fluorescent protein (GFP) gene driven by the murine stem cell virus (MSCV) U3 promoter [36], where the woodchuck hepatitis response element (wPRE) was replaced with the truncated form, oPRE as previously described [37] (vector termed pLVV.GFP.oPRE). A lentiviral vector expressing an anti-MART-1 TCR was based on the previously reported [38] vector and is termed pLVV.coDMF5.oPRE. The human CD62L gene (GenBank accession: AJ246000) was isolated from total RNA of *in vitro* cultured PBLs by reverse-transcription-PCR using primers, 5′-ggtacctgccggcgcgccgccgccatgggcatatttccatggaaatgtcagagc, and 3′- gccgtcgacttaatatgggtcattcatacttctcttg. The PCR products were purified and digested with Asc I and Sal I and ligated into corresponding sites of pLLV.GFP.oPRE, to produce pLVV.hCD62L.oPRE. The shedding resistant mutant of human CD62L dK-S [4] was made with primers (5′-cctagtccaatatgtcaaatgattaaggagggt, 3′-atcaccctccttaatcatttgacatattggact) by site-directed mutagenesis kit (Stratagene, La Jolla, CA). All the constructs are confirmed by restriction enzyme digestion and DNA sequencing.

The production of lentiviral vector supernatants has been previously described in detail [34], [38], [39]. Viral vector particle concentration was estimated using a p24 ELISA (ZeptoMetrix, Buffalo, NY) and was either used directly or stored at −80°C.

PBLs were transduced with the vector by spinoculation method using 6-well culture plates. Briefly, 10^6^ PBLs were washed twice with PBS and pre-incubated with Dynal anti-CD3/CD28 beads (Invitrogen, Carlsbad, CA) (cells to beads ratio 1∶2) for 10 min at room temperature and followed by overnight incubation. Next day, 5 ml vector plus 1 ml AIM-V (with 10% FBS, IL-2 300 IU/ml) were applied in the presence of 10 µg/ml protamine sulfate, and the plates were centrifuged at 1000 g, 32°C for 2 h. Six hours posttransduction, the cells were transferred to 75 CM^2^ flasks for continued culture in AIM-V containing 5% human serum, 300 IU/ml IL-2. Transduction of CD8+ T cells was accomplished using OKT3-coated plates as previously described in detail [40].

### FACS analysis and sorting

Cell surface expression of CD3, CD8, CD62L, CD107a and CD45RO was measured using fluorescein isothiocyanate (FITC), allophycocyanin (APC), phycoerythrin (PE), PE-Cy7, and APC-Cy7-conjugated antibodies (BD Biosciences, San Jose, CA). Custom designed MART-1: 27–35 tetramer was used (iTAg MHC Tetramer, Beckman Coulter, Fullerton, CA), to detect TCR gene-transduced cells. For live cell staining, cells were washed with FACS buffer (PBS containing 2% FBS), and antibodies described above were added directly into cells (10^6^/ml) followed by 30 min incubation at 4°C, the cells were washed twice with FACS buffer. To gate out of dead cells, propidium iodide (Sigma-Aldrich, Saint Louis, MO) in PBS was added to samples in a final concentration 5 µg/ml before FACS analysis. Interferon-gamma (IFN-γ) was measured using intracellular staining kit with reagents and protocols as directed by the manufacturer (BD Biosciences). Briefly, cells were resuspended in fixation/permeabilization solution for 20 min at 4°C, then washed the cells twice using perm/wash buffer followed by the addition of fluorochrome-conjugated antibodies followed by 30 min incubation at 4°C. No propidium iodide staining was performed for samples subject to intracellular staining. Immunofluorescence staining was analyzed as the relative log fluorescence of live cells, determined using a FACscan flow cytometer (BD Biosciences). A combination of forward angle light scatter and propidium iodide staining was used to gate out the dead cells, and 1×10^5^ cells were analyzed. FACS analysis was performed by the staff of the FACS core facility at Surgery Branch/National Cancer Institute, Bethesda MD. Six-color analysis was carried out on a Canto I or Canto II instrument (BD Biosciences) with automatic compensation. Cultured T cells were sorted using a FACSAria cell sorter (BD Biosciences). Briefly, T cells were labeled with CD62L, CD45RO and CD8, subsets of CD62L+/CD45RO+ and CD62L−/CD45RO+ were sorted. FACS data was analyzed using FlowJo 8.1.1 software (FlowJo, Ashland, OR).

### Purification of CD8 T cells and CD62L positive cells

CD8+ and CD62L+ Microbeads (Miltenyi Biotec) were used for purification of CD8+ T cells from PBMCs and CD62L+ TIL from the transduced JKF6 line, following instructions as detailed from the manufacturer.

### Measurement of lymphocyte reactivity

The ability of transduced JKF6 to lyse HLA-A2+/MART-1 melanoma cells was evaluated using a ^51^Cr assay as described [41]. Briefly, 10^6^ tumor cells were labeled for 1 h at 37°C with 100 µCi of ^51^Cr (Amersham Biosciences, Pittsburgh, PA) in 2 ml of media. Labeled target cells (2×10^3^) were co-cultured with effector cells at the ratios indicated in the figures for 4 h at 37°C in 0.15 ml of complete medium. Harvested supernatants were counted using a MicroBeta TriLux instrument (Perkin Elmer, Waltham, MA). Each data point was determined as the mean of quadruplicate wells. The percentage of specific lysis was calculated as indicated in figure legend. Determination of the amount of CD62L shedding was performed as follows. The central memory enriched CD8+ T cells expressing anti-MART-1 TCR were co-cultured with melanoma lines (1×10^6^ each) in 14 ml round-bottom polypropylene tubes for 4 h (volume,1 ml). Cells were centrifuged (800×g 10 min) and supernatant were collected, and the cell pellet was lysed using RIPA Buffer. The amount of CD62L in supernatant and in cell lysates was determined by ELISA (R&D Systems, Minneapolis, MN).

## Results

### The loss of CD62L is tumor antigen specific and is associated with CD107a assembly

Using lentiviral vector transduction to introduce a tumor antigen-specific TCR into human open repertoire T cells readily generates anti-tumor T cells that have the potential for use in adoptive immunotherapy [34], [38]. As previously reported, the bulk of these short-term cultured T cells have a central memory-like phenotype defined by the expression of cell surface markers CD45RO and CD62L [35] [in FACS analysis these populations are defined as follows; CD45RO+/CD62L− (Tem), CD45RO+/CD62L+ (Tcm) or CD45RO^low^/CD62L+ (Tn), and CD45RO−/CD62L− (TemRA, effector-memory CD45RA+)]. To determine the lytic potential of these *in vitro* generated CD8+ T cells, we utilized T cells genetically engineered with an anti-MART-1 TCR as effector cells and melanoma lines as target cells. In this model, lytic potential is measured by the cell surface expression of the degranulation marker CD107a [31]. CD107a is lysosomal-associated membrane protein 1 (LAMP1), and it is a component of lysosomal-associated vesicles that contain granzyme B and perforin [31].

**Figure 1:**
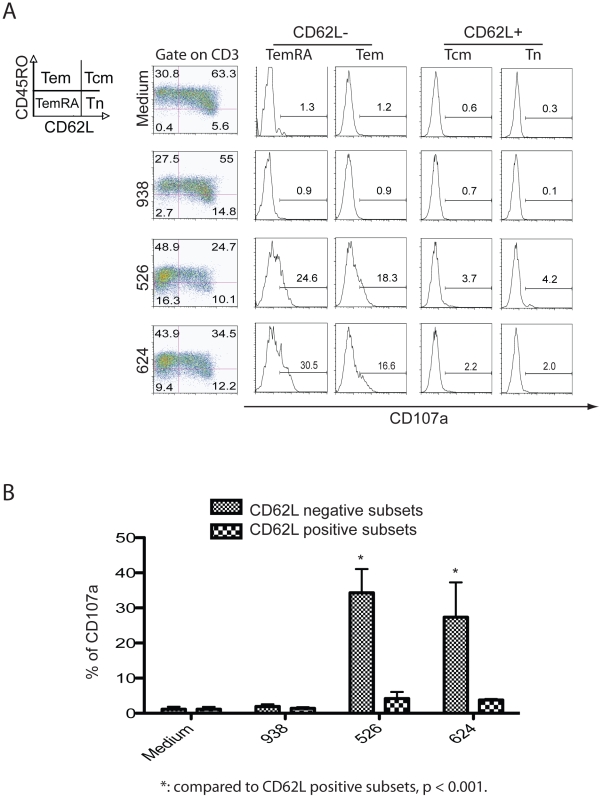
The loss of CD62L is specific and correlates with CD107a surface expression. **A.** MART-1 TCR vector-transduced T cells were co-cultured with melanoma lines 526, 624 (both HLA-A*0201 positive) and 938 (HLA-A*0201 negative) for four hours and then analyzed by FACS. The first column on left, the cells were plotted using differentiation markers CD45RO and CD62L into 4 subsets, the expression of CD107a on each subset was denoted in the histograms to the right. **B.** Based on data from three independent experiments, the expression of CD107a after co-culture with melanoma lines was determined and mean% ± stdev plotted. Student t-test was used for statistical analysis. The fluorophore conjugated antibodies used in this analysis were CD107a FITC, CD62L PE, CD45RO APC, CD3 APC-Cy7 with propidium iodide staining.

When MART-1 TCR engineered effector T cells were co-cultured with HLA-matched melanoma cell lines 526 and 624 antigen-specific loss of CD62L expression was observed in the Tcm quadrant (figure 1A, left column). We next looked for the expression of CD107a in the different T cell populations (figure 1A, histograms). CD107a formation was readily observed in the CD62L− populations of effector memory (Tem) and effector memory CD45RA+ (TemRA); but was minimal expressed in the CD62L+ populations of central memory (Tcm) and naïve cells (Tn). As expected, CD107a expression was not observed in T cells not exposed to targets or exposed to the HLA mismatched tumor line 938 (figure 1). This was a reproducible finding and statistically significant (Figure 1B, p<0.001).

**Figure 2:**
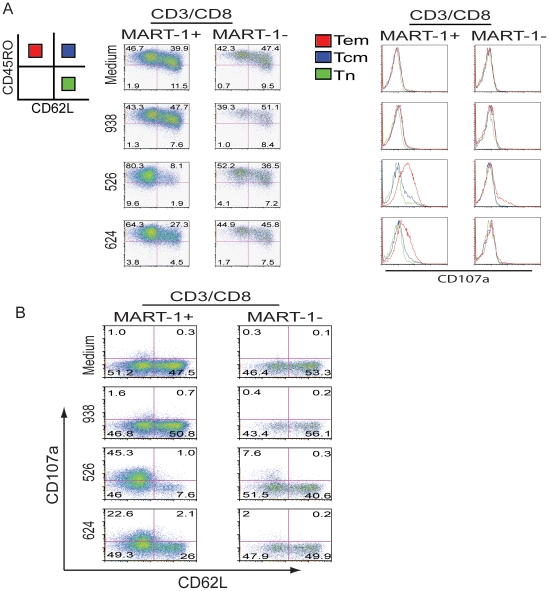
The loss of CD62L is tumor antigen specific and the expression of CD107a occurs on CD62L negative cells. **A.** TCR transduced cells were cultured as described in methods. Shown is the FACS analysis, where cells were gated on CD3/CD8/MART-1+ or CD3/CD8/MART-1−, and then plotted using markers CD45RO and CD62L (left two column plots). The expression of CD107a on each group was then determined and separately plotted in the histograms on right corresponding to column data on left. In each histogram the expression of CD107a on each subset was overlaid reflecting Tem, Tcm and naïve populations. Red (CD45RO+CD62L−) indicates Tem, blue (CD45RO+CD62L+) indicates Tcm, and green (CD45RO−CD62L+) indicates Tn population. **B.** The cells were processed as described above. By FACS analysis, the cells were gated on CD3/CD8/MART-1+ or CD3/CD8/MART-1−, then plotted using markers CD107a and CD62L in dot plots. The fluorophore conjugated antibodies used in this analysis were CD107a FITC, CD62L PE, CD45RO APC, CD3 APC-Cy7 with propidium iodide staining.

To confirm that this observation was tumor-antigen specific, the experiment was repeated with an additional PBL donor and analysis of marker gene expression further refined by gating on MART-1 TCR gene-engineered cells (Figure 2). By gating on CD3/CD8/MART-1+ T cells following co-culture, we detected a more pronounced loss of CD62L expression compared to MART-1 tetramer negative cells (Figure 2A, compare 1^st^ and 2^nd^ columns). We observed a concomitant increase in CD107a formation in the MART-1 TCR+ population upon co-culture with MART-1+/HLA matched tumor lines (Figure 2A, compare 3^rd^ and 4^th^ columns). Again the expression of CD107a is primarily observed in the Tem population. These observations were repeated in two additional donors (data not shown). To determine the relation of CD107a and CD62L on a per cell basis, co-cultured cells were also analyzed and presented as dot plots (Figure 2B). Consistent with figure 1, we observed the expression of CD107a predominantly in the CD62L null population. Furthermore, the expression of CD107a was inversely correlated with the cell surface expression of CD62L (Figure 2B).

### The loss of CD62L is due to antigen induced shedding from T cells

**Figure 3:**
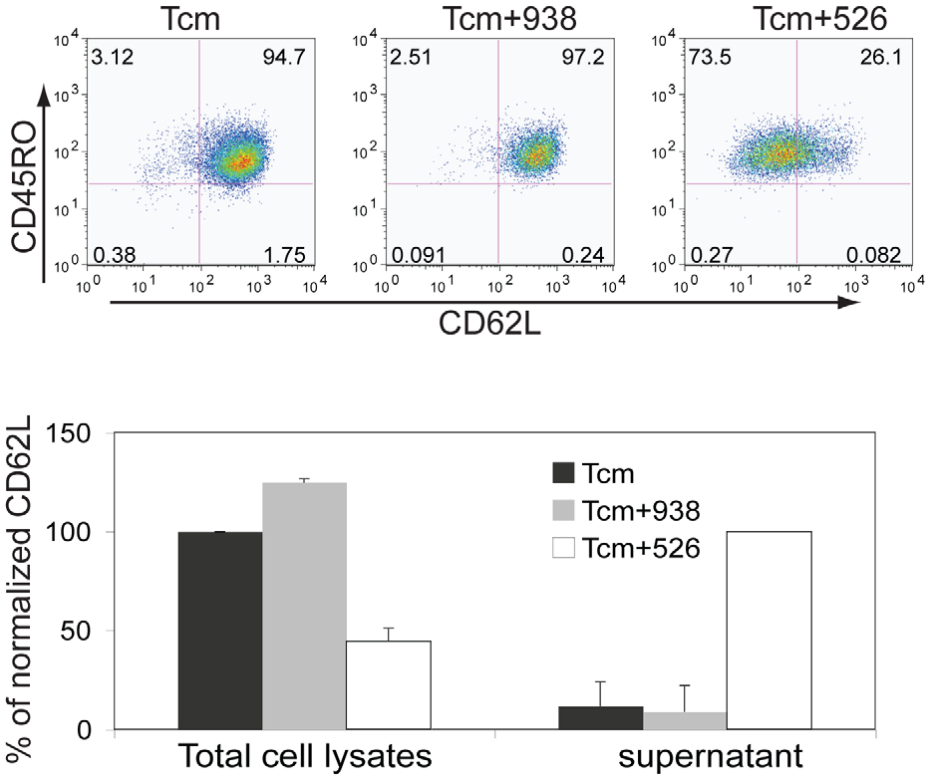
The loss of CD62L is due to antigen induced shedding in T cells. **Top.** MART-1 TCR transduced cells were gated on the CD8+ and CD45RO+/CD62L+ (Tcm) population and sorted by FACS. The sorted Tcm cells were co-cultured with melanoma lines for 4 h. Culture media (supernatant) was collected and the cells stained for FACS analysis. Shown in the top plots is the expression of CD45RO and CD62L in unmanipulated Tcm cells, Tcm cells cultured with melanoma line 938 (HLA-A0201−), and Tcm cells cultured with melanoma line 526 (HLA-A0201+). The numbers in each quadrant represent the percent of cells in that quadrant. The fluorophore conjugated antibodies used in this analysis were CD107a FITC, CD62L PE, CD45RO APC, CD3 APC-Cy7 with propidium iodide staining. **Bottom.** The amount of CD62L in the cell media supernatant and total cell lysates was measured by ELISA. The amount of CD62L in the total cellular lysate in the Tcm group was set as 100% for comparison to the other lysate values (left side bars). For the cell supernatant determinations, the amount of soluble CD62L in the supernatant from the Tcm+526 group was set as 100%, for comparison to other values (right side bars). Data shown are the normalized values ± stdev for three independent determinations.

**Figure 4:**
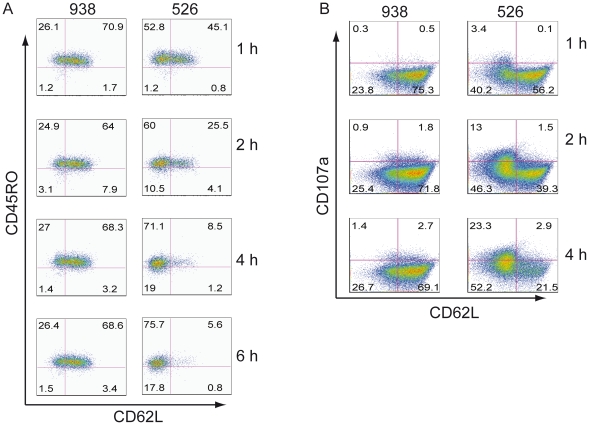
The dynamics of CD62L shedding is inversely correlated with the expression of CD107a. **A.** The dynamics of CD62L shedding in CD8+/CD62L+ population was followed over 6hr. CD8+ MART-1 TCR transduced Tcm cells were sorted by FACS as in figure 3. These sorted cells (CD45RO+/CD62L+) were co-cultured with melanoma lines 526 (HLA-A0201+) and 938 (HLA-A0201−) for the times indicated and analyzed for CD45RO and CD62L. Column on left (co-cultured with 938) and on right (co-cultured with 526). The numbers in each quadrant represent the percent of cells in that quadrant. **B.** The expression of CD107a correlates with the shedding of CD62L in CD8+/CD62L+ population. CD8+ MART-1 TCR transduced T cells from a different donor were co-cultures as described in methods. The cells were gated on CD3/CD8/MART-1+ and then plotted for CD107a and CD62L expression. The samples were collected and stained at the times indicated on right. The fluorophores conjugated antibodies for this analysis were CD107a FITC, MART-1 PE, CD62L APC, CD45RO PE-Cy7, CD8 APC-Cy7 and propidium iodide staining.

MART-1 TCR transduced T cells were sorted for the CD8+ Tcm population and then co-cultured with melanoma lines for 4 h (Figure 3). At the completion of the co-culture, the phenotype of the cells was determined and we again observed the loss of CD62L expression from the Tcm cells only in the co-culture with MHC matched tumor line 526. The shedding of CD62L from T cells following mitogen stimulation is well studied [7], [42]. To determine if the loss of CD62L expression on these TCR gene–engineered T cells was similarly caused by shedding, we assayed for the presence of CD62L in both total cell lysates and in cell culture media. As measured by ELISA, we found there was a reduced level of CD62L in total cell lysates in the MHC matched 526 group, with a concomitant increased level of soluble CD62L in the cell culture media from the same group (Figure 3, bottom), suggesting that CD62L is actively shed into the culture upon antigen encounter. To determine the dynamics of this shedding process, we repeated the co-culture of the sorted CD8+ Tcm enriched population with melanoma cells and found that shedding was detected as early as 1 h after encountering tumor antigen (Figure 4A). Moreover, we could detect the shedding as early as 15 min after tumor antigen encounter (data not shown). The shedding progressed over time and within 4–6 h, shedding reached its peak (Figure 4A). In another independent experiment, we confirmed the dynamics of CD62L over time, and again observed the concomitant increase of surface CD107a expression following the shedding of CD62L (Figure 4B).

### Introduction of CD62L shedding resistant mutants downgrades T cell anti-tumor activity

**Figure 5:**
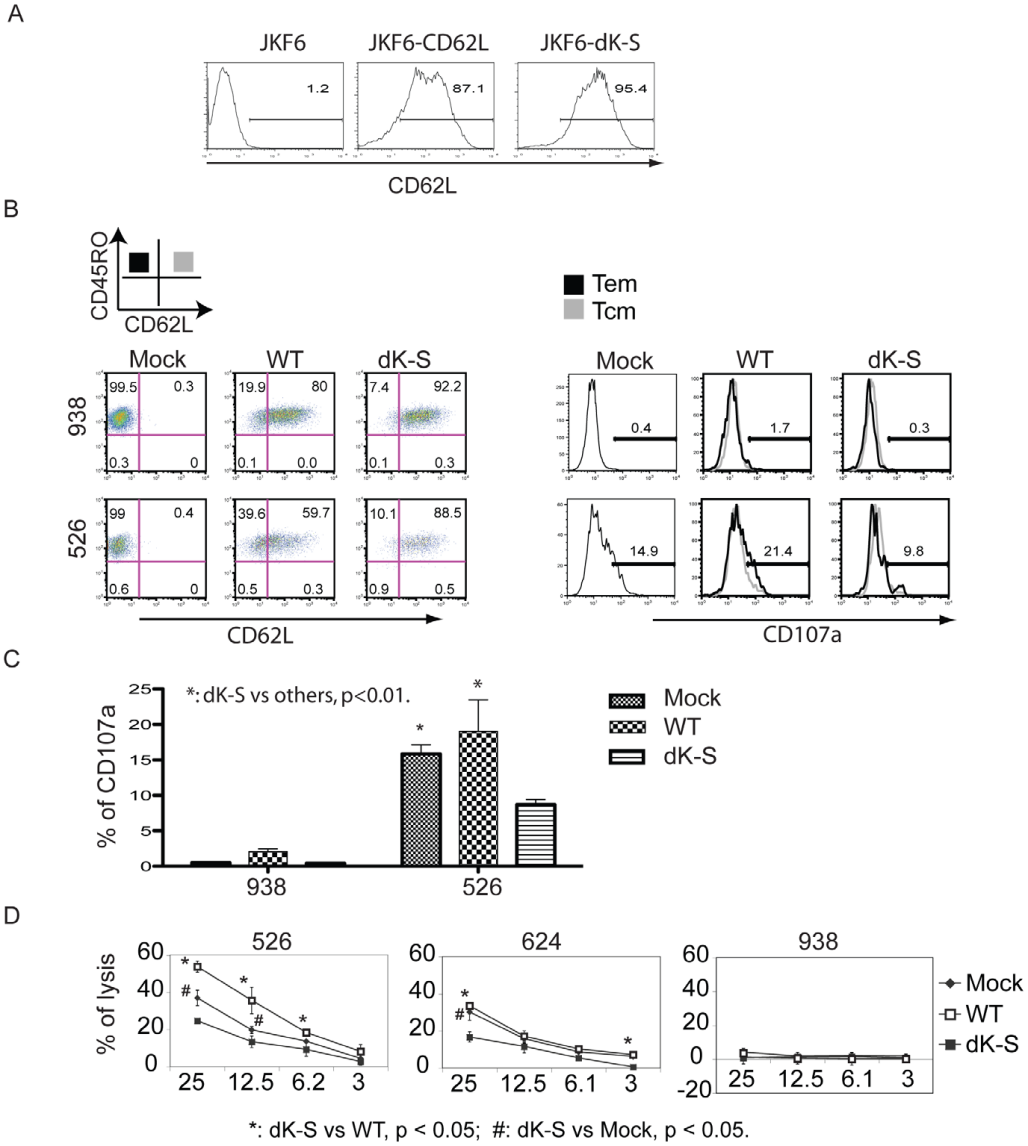
Introduction of shedding resistant CD62L affects the surface expression of CD107a. **A.** TIL line JKF6 was transduced with lentiviral vector harboring human CD62L and its shedding resistant mutant dK-S. JKF6 cells expressing CD62L and the dK-S mutant were enriched using CD62L beads and analyzed for CD62L expression by FACS. **B.** JKF6 gene engineered cell lines were co-cultured with melanoma lines 526 (HLA-A0201+) and 938 (HLA-A0201−) analyzed for CD45RO and CD62L expression (Mock, mock transduced cells; WT, CD62L transduced JKF6; dK-S, JKF6 transduced with shedding resistant mutant). On left, FACS plots from co-cultures of different groups; on right, overlayed histograms of the expression of CD107a in Tem and Tcm subsets. Solid line (CD45RO+CD62L−) indicates Tem, semi-solid line (CD45RO+CD62L+) indicates Tcm. **C.** Quantification of CD107a expression from triplicate determinations was calculated and plotted as % CD107a+ (mean ± STDEV). Statistical analysis using t test; *: dK-S vs mock and WT, p<0.01. **D.** The lytic activity of JKF6 lines expressing CD62L and its mutant were determined by co-culture with ^51^Cr-labeled melanoma lines at the indicated ratios. The percent cell lysis was calculated using the formula ((specific release-spontaneous release)/(total release-spontaneous release))×100. Results representing mean of triplicate cultures were plotted. *: dK-S vs WT, p<0.05; #: dK-S vs Mock, p<0.05. The t-Test with two samples assuming equal variances was used for statistical analysis. The fluorophores conjugated antibodies for this analysis were CD107a FITC, MART-1 PE, CD62L APC, CD45RO PE-Cy7, CD8 APC-Cy7 and propidium iodide staining.

The shedding of CD62L from T cells is an active process mediated by a membrane bound protease, ADAM17. We prepared lentiviral vectors harboring human wild type CD62L and a shedding resistant mutant of CD62L, dK-S (a 7 amino acid deletion covering K280-S286) [4]. CTL line JKF6 is a long-term cultured, anti-MART-1 reactive tumor infiltrating lymphocyte (TIL) that does not express CD62L. We transduced JKF6 with lentiviral vectors expressing the wild type CD62L and the dK-S mutant, and selected these cells for uniform CD62L expression (Figure 5A). The purified JKF6 cells constitutively expressing wild type CD62L and its mutant were then co-cultured with melanoma lines.

Following co-culture, transduced cells were analyzed using CD45RO and CD62L, and similar to our observations with PBL, we observed a loss of CD62L expression (presumptively by shedding) following co-culture of the wild-type CD62L gene-engineered JKF6 cell line with HLA matched melanoma lines (Figure 5). The loss of CD62L expression was abolished in the JKF6 cells expressing the CD62L shedding resistant mutant dK-S (Figure 5B). As we observed in PBL, the detection of CD107a was inversely related to the shedding of CD62L (Figure 5B, histograms on right). We observed an increase of CD107a in the cells engineered with the wild-type CD62L, while there was reduced CD107a formation in JKF6 cells engineered with the shedding resistant mutant (Figure 5B). Overall, there was approximately a 50% reduction in the CD107a surface expression (p<0.01) in JKF6 cells engineered with the dK-S mutant CD62L gene (Figure 5C). To determine whether the reduction in CD107a formation was linked to the ability of JKF6 to lyse melanoma cells, the engineered cells were assayed in a standard 4 h 51Cr-release assay (Figure 5D). Results of this experiment demonstrate that the lytic activity of the JKF6 cells expressing the shedding resistant mutant dK-S was significantly downgraded (p<0.05) compared to cells engineered with the wild-type CD62L or mock transduced cells.

**Figure 6:**
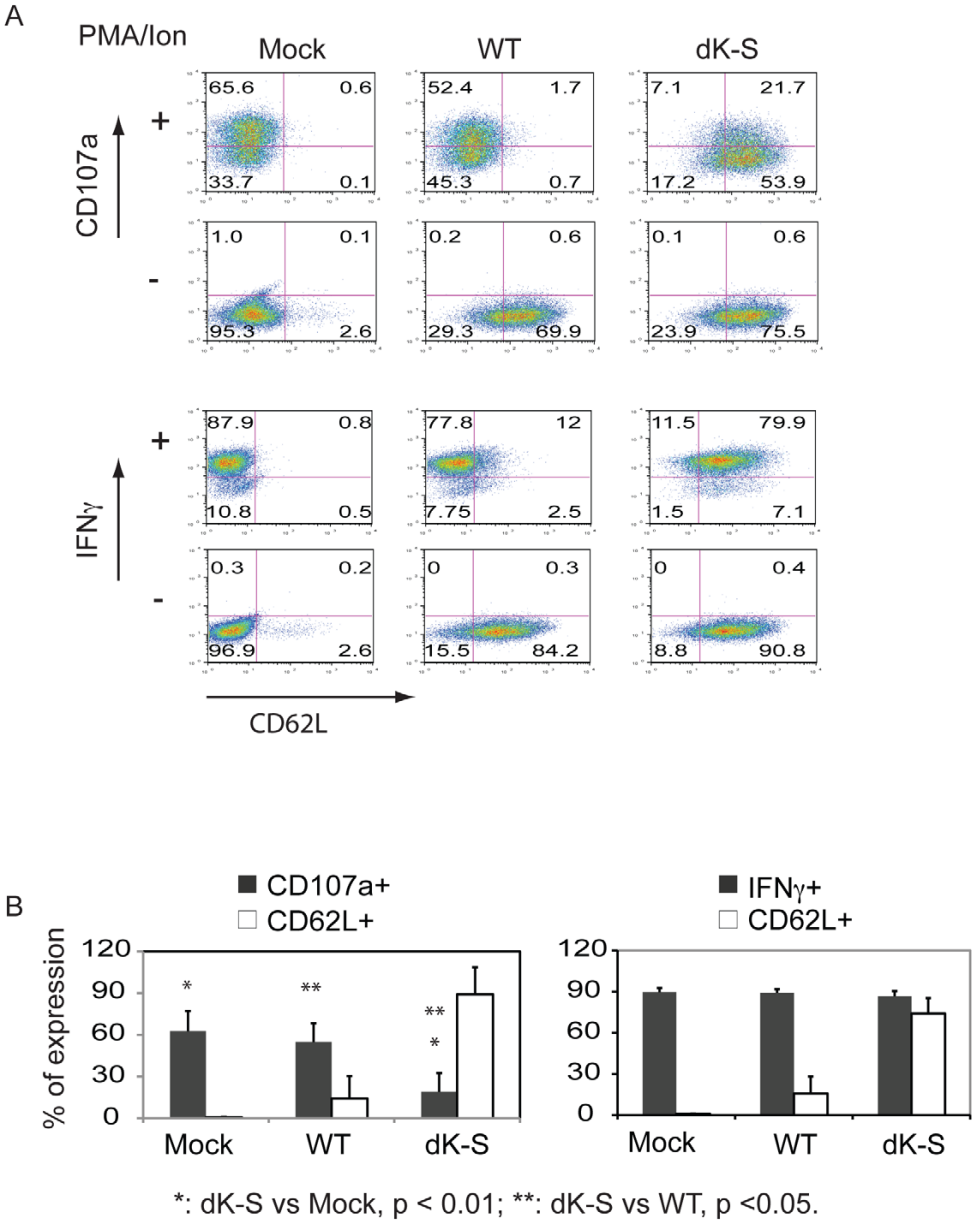
Introduction of shedding resistant CD62L into JKF6 hinders CD107a expression following non-specific activation. **A.** JKF6 lines expressing CD62L and mutant dK-S were activated using PMA/Ionomycin (PMA/Ion) for 4 h, and the surface expression for CD107a and IFN-γ was evaluated by FACS. One representative result from three independent experiments is shown (Mock, mock transduced cells; WT, CD62L transduced JKF6; dK-S, JKF6 transduced with shedding resistant mutant). For CD107a live cell staining, the fluorophores conjugated antibodies were CD107a FITC, CD62L APC, CD3 PE and propidium iodide staining; For intracellular staining of IFN-γ, the fluorophore conjugated antibodies were IFN-γ FITC, CD62L APC, CD3 PE, without propidium iodide staining. **B.** Plot of the percent positive cells for CD107a and IFN-γ on the surface of JKF6 lines expressing wild type and shedding resistant CD62L mutant. The mean ± STDEV from three independent experiments is displayed, and t-test was used for statistical analysis.

To determine if the link between CD62L expression and CD107a mobilization was dependent on TCR-HLA-A2 interactions, we bypassed this antigen-specific activation using PMA/Ionomycin (Figure 6). When the engineered JKF6 cells were non-specifically activated, there was a significant inhibition in the ability of cells to express CD107a on the cell surface if they were engineered with the shedding resistant dK-S mutant CD62L gene (28.8%) compared to mock-transduced (66.2%) and wild-type (54.1%) engineered cells (Figure 6A). This observation was not due to a lack of the ability for PMA/Ionomycin to stimulate these cells, as production of the effector cytokine IFN-γ was not affected (Figure 6A). The overall reduction in CD107a surface expression was about 70% compared to mock-transduced and wild type CD62L engineered cells and this was statistically significant (Figure 6B).

## Discussion

The CD62L knockout mouse model indicates that CD62L plays an essential role in lymphocyte homing to lymphoid tissues and sites of inflammation, and that CD62L shedding from antigen-activated T cells prevents their reentry into peripheral lymph nodes [7]. However, transgenic mice expressing cleavage-resistant CD62L mutants delay, but do not negate effective immune response to viral infections [9], suggesting that lymphocyte trafficking is only part of a successful anti-viral response. It has been reported that the shedding of CD62L is primarily mediated by the metalloprotease ADAM17. Many leukocytes express ADAM17, and a number of ADAM17 substrates are immunologically important proteins including; TNF-α, its receptors TNFRI and TNFRII, and CD62L [6], [43]. CD62L is also present at relatively high levels in the serum of normal individuals, and it has been postulated that this may direct leucocytes to sites of inflammation [44]. Our data suggest CD62L is not simply a homing molecule, rather its' shedding after activation may play a novel role in determining the acquisition of lytic function as measured by the cell surface expression of CD107a, a marker of T cell degranulation [21], [30], [31], [32].

In this study, we generated an *in vitro* model illustrating the dynamics of CD62L expression on human cytotoxic T lymphocytes following encounter with tumor antigen. CD62L shedding was initiated within minutes after CTL encounter with tumor antigen and reached its maximum level at 4–6 h post-activation, consistent with reports for murine T cells [42]. CD62L shedding is not limited to T cells but is also found in cells of the innate immune system. In resting neutrophils, CD62L is constitutively expressed at high levels, and essentially all molecules are shed within minutes following neutrophil activation [45]. The difference in the rate of shedding between T cells and neutrophils might represent the activity of ADAM17 on their surfaces, or their sites of activation. While T cell activation occurs within the lymph node, the activation of neutrophils (which is not antigen-specific) occurs rapidly at sites of infection, which presumptively facilitates the eradication of pathogens. However, the activation of T cells (a member of the adaptive immune system) occurs more slowly via antigen presenting cells (APC) within lymph nodes. The slow shedding of CD62L from the surface of T cells might provide a protective factor for lymphoid tissues, by allowing activated T cells to migrate out of the lymph nodes as they become fully activated. Our data support this hypothesis in that the surface of CD107a occurred only in T cells after shedding of CD62L.

Since the putative primary cleavage site (K283-S284) of human CD62L was identified, we cloned wild type and a shedding resistant mutant dK-S [4] into lentiviral vectors to directly test for an association between CD62L and CD107a. In all of our assays, the dK-S mutant was almost completely resistant to activation induced shedding. The cytoplasmic tail of CD62L is highly basic and consists of only 17 amino acids that have been reported to regulate shedding, microvillus positioning and the tethering/rolling [46]. Specifically, the cytoplasmic tail of CD62L has been reported to interact with at least three different proteins [47] including calmodulin, α-actinin (a member of the ezrin/radixin/moesin (ERM) family of membrane-cytoskeleton cross-linkers), and protein kinase C isoenzymes. Disruption of these interactions may reduce the shedding [48] or inhibit tethering/rolling efficiencies in vitro [49].

In this study, we found the shedding of CD62L from the surface of T cells was antigen specific, and CD107a surface expression could only be detected in cells that had shed CD62L. Moreover, when we introduced a shedding resistant mutant of CD62L into T cells, this not only blocked CD62L shedding but also affected the surface expression of CD107a and this correlated with a downgraded ability of these cells to lyse targets. Thus our data suggest that there is a link between the shedding of CD62L and the acquisition of T cell lytic ability. As the cytoplasmic tail of CD62L interacts with molecules such as α-actinin, and α-actinin can interact with the cytoskeleton, we hypothesize that the abolishment of CD62L shedding from the T cell surface could ultimately affect the cytoskeleton structure, which in turn may disrupt the mobilization of cytotoxic granules to the cell surface (which is measured by the surrogate maker CD107a) and release of perforin and granzyme B to initiate target cell lysis.

It is interesting to note that, in the case of *ex vivo* cultured murine lymphocytes used for adoptive immunotherapy to treat B16 melanoma, extended culture periods result in loss of CD62L expression and this is correlated with decreased effectiveness *in vivo* [50]. This observation has been used to support the hypothesis that terminally differentiated T cells are less effective anti-cancer cells [50]. Our date would further suggest a potential biochemical link between the loss of CD62L and decreased effector functions. Clearly the immune system maintains a balance between T cell homing to sites of infection/inflammation and effector function, and our data suggest that one of the main trafficking molecules, CD62L, may also be involved with the acquisition of effector cell function.
